# Removing a Foreign Body from the Œsophagus

**Published:** 1883-07

**Authors:** 


### IV.—A NOVEL WAY OF REMOVING A FOREIGN BODY FROM THE ŒSOPHAGUS.

REPORTED FROM DR. NEUMAN'S CLINIC HELD AT HIS PRIVATE INFIRMARY.

The Doctor was called to see a bay horse which was choking. The history was that he had been eating carrots. Treatment:

He was unable to reach the foreign body with his hand. He gave a dose of oil at once, but it failed to dislodge the body. The horse's mouth was then opened, and held open by a mouth speculum. Then a common water hose, which was at hand, was passed through the speculum down the œsophagus until the foreign body was reached. [Whether the hose was used simply as an œsophagal bougie, or the force of the hydrant was called into play, was not stated by the clinical reporter, but we inferred that it was flooded down.–[Ed.

